# Assessing the ecotoxicological effects of pesticides on non-target plant species

**DOI:** 10.1007/s10661-025-14532-2

**Published:** 2025-08-27

**Authors:** Iwona Gruss, Paulina Bączek, Irmina Ćwieląg-Piasecka, Szymon Jędrzejewski, Joanna Magiera-Dulewicz, Kamila Twardowska

**Affiliations:** 1 Department of Plant Protection, Wroclaw University of Environmental and Life Sciences, Wroclaw, Poland; 2 Institute of Soil Science, Plant Nutrition and Environmental Protection, Wroclaw University of Environmental and Life Sciences, Wrocław, Poland

**Keywords:** Phytotoxicity, Pesticide mixtures, Synergistic effects, Toxicity assessment

## Abstract

Over the past two decades, pesticide use has increased globally, with over 91% of crops in the EU being treated. While pesticides improve crop yields, they also pose environmental risks. They can affect non-target plants by inhibiting germination, suppressing growth, and impairing photosynthesis. Pesticide drift, runoff, and soil contamination may expose wild plant communities to sublethal concentrations, potentially disrupting biodiversity and ecosystem stability. This study examined the effects of commonly used pesticides in European agriculture on non-target plant species. Six active ingredients—acetamiprid, pirimicarb, glyphosate, fluroxypyr, azoxystrobin, and tebuconazole—were tested individually and in mixtures at realistic field concentrations. Toxicity was assessed in *Sinapis alba*, *Lepidium sativum*, and *Sorghum saccharatum*. Results showed that fluroxypyr (a herbicide) was consistently the most phytotoxic compound. All tested pesticide mixtures produced stronger phytotoxic effects than their components. Among the three models used to predict mixture toxicity—additive, dominance, and multiplicative—the additive model most accurately reflected the observed responses, assuming that the total effect equals the sum of the effects of individual substances. However, the model did not fully explain the enhanced toxicity observed in certain combinations, suggesting potential synergistic interactions. These findings underscore the need for further research on the ecological impact of pesticide mixtures.

**Graphical Abstract:**

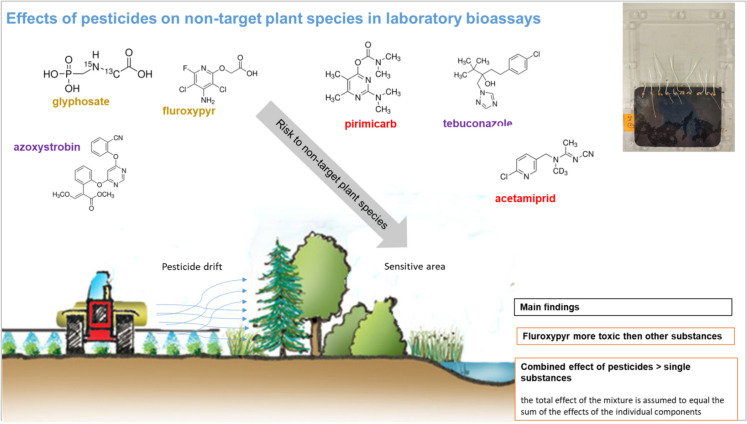

**Supplementary Information:**

The online version contains supplementary material available at 10.1007/s10661-025-14532-2.

## Introduction

Pesticides play a crucial role in modern agriculture, as they protect crops from pests, weeds, and diseases, thereby ensuring high yields and food security (Safdar et al., 2022). However, their widespread use has raised significant concerns about unintended ecological consequences. It exerts substantial effects on non-target terrestrial ecosystems, impacting plants, arthropods, and other organisms (Beringue et al., 2024). Herbicides are designed to control weeds, but these substances can also reduce plant biomass and biodiversity, indirectly affecting arthropod communities and bird populations (Mansuripur, 2011). Insecticides, while targeting harmful pests, also negatively impact beneficial insects such as pollinators and natural predators, leading to imbalances in food webs (Samanta et al., 2023). Meanwhile, fungicides widely used to prevent crop diseases disrupt soil microbial communities, harm invertebrates, and accumulate in the food chain, causing sublethal effects on various species (Meyer et al., 2024). Fungicides applied to almond crops have been shown to reduce the richness and diversity of beneficial nectar-inhabiting fungi, particularly *Metschnikowia* species, which play a role in plant-pollinator interactions (Schaeffer et al., 2017). Commercial fungicide formulations may contain ingredients that are as toxic or more toxic than the active ingredients, potentially underestimating their effects on non-target species like earthworms (Jorge-Escudero et al., 2022). Studies on earthworms have shown that fungicides can cause sublethal effects, including reduced progeny, and some species, like *Glossoscolex rione* may be more sensitive than commonly tested species like *Eisenia fetida* (Jorge-Escudero et al., 2022). Exposure to the fungicide folpet at realistic field rates can induce lethal and sublethal effects on juvenile European common frogs (Adams et al., 2020). Fungicide exposure can increase amphibian susceptibility to the fungal pathogen *Batrachochytrium dendrobatidis*, potentially exacerbating amphibian mortality.

Not only do pesticides have direct toxic effects, but they also affect complex ecological interactions, including impact on non-target organisms, development of resistance, and pesticide residues entering the food chain (Wan et al., 2025). While habitats near cultivated fields are not the intended targets during pesticide application, they can still be severely impacted. Such exposure can happen through direct overspray (especially with aircraft applications), spray drift from treated crops to nearby wildlife areas, or runoff and wash-off (Kumar et al., 2021). Pesticides may drift up to 500 m downwind when applied by aerial equipment (Albaseer et al., 2025). Therefore, margins, hedgerows, woodlots, riparian habitats, and wetlands are important refuges for annual and perennial plant species and habitats of wildlife may be negatively impacted by widespread pesticide application (Ferreira, 2024).

According to the specific effects of pesticides on non-target plant species, they may depend on pesticide type, environmental conditions, and plant characteristics (Boutin et al., 2014) and include plant survival, growth, and reproduction (Karthikeyan et al., 2004). These effects can occur through various mechanisms, such as changes in the plants’absorption, translocation, and metabolism (Lushchak et al., 2018). The effects of other pesticide groups (then herbicides) on non-target plants are poorly explained. The sensitivity of non-target plant species to herbicides varies among plant species, with some exhibiting tolerance while others show high susceptibility (Florencia et al., 2017; Karthikeyan et al., 2004). Some plant species may develop detoxification mechanisms to overcome pesticide exposure, but the long-term effects on biodiversity and ecosystem functioning remain a concern (Ferreira et al., 2023; Karthikeyan et al., 2004). Indirect impacts of herbicide toxicity on non-target plant species include pest outbreaks due to the elimination of natural enemies, declines in pollinator populations caused by herbicide-induced loss of floral diversity, and disruptions in aquatic ecosystems due to changes in species composition and nutrient cycling (Brühl & Zaller, 2021; Sánchez-Bayo, 2021). Another concern is the persistence and accumulation of herbicides in soil, which can lead to prolonged exposure to non-target plants. Further, raises concerns about bioaccumulation in plant tissues, food chain contamination, and potential human health risks (Pullagurala et al. 2022; Pririti et al., 2024). Also, some pesticides accumulate in plant tissues (most frequently in roots) over time, potentially causing chronic toxicity that affects growth, reproduction, and overall health (Kamal et al., 2020).

Nonetheless, pesticide residues, often composed of several substances approved for agriculture, are commonly present in soils at varying levels. Their ecological impact remains poorly understood (Silva et al., 2019). While current EU regulations require evaluating combined effects for plant protection products, guidance for assessing mixture toxicity varies across different organism groups and is still developing for terrestrial organisms (Panizzi et al., 2017). Certain pesticides, particularly herbicides and fungicides, can interact synergistically or antagonistically, amplifying their toxic effects on non-target species (Panizzi et al., 2017). For example, pesticide combinations may weaken plant defences, increasing susceptibility to pathogens and environmental stress (Cedergreen et al., 2007). The toxicological effects of pesticide mixtures can be independent, additive, or interactive, with interactions occurring in spray solutions, within plants, or in soil (Barrett, 2018). Understanding these complex interactions is crucial for accurate risk assessment, as the combined effects of low-dose pesticide mixtures in the environment remain largely unknown (Liu & Sayes, 2024).

There are many approaches to measuring a particular substance’s toxic effect on plants. One of them is the Phytotoxkit method, which is a rapid and cost-effective approach for assessing different contaminants’phytotoxicity compared to traditional plant toxicity tests (Blok et al., 2009; Van der Vliet et al., 2012). These standardised methods use transparent containers with filter paper to measure seed germination and seedling growth of various plant species exposed to potentially toxic substances (Blok et al., 2009). Bioindication using seed germination and seedling growth parameters can effectively assess pesticide phytotoxicity, with studies showing a direct relationship between pesticide exposure and inhibition of plant development (Petruk et al., 2021). This method is not widely used for pesticide risk assessment, but it may help to understand how pesticide combinations affect non-target plant species.

This study evaluated the effects of six active pesticide ingredients, applied individually and in mixtures, on the non-target plant species *Lepidium sativum*, *Sinapis alba*, and *Sorghum saccharatum* under controlled laboratory conditions. It was hypothesized that herbicides applied at field-realistic concentrations would exert the strongest phytotoxic effects on plant growth and development. Furthermore, based on previous studies reporting interactions among similar pesticides and their combined effects on non-target plants ((Cedergreen et al., 2007; Panizzi et al., 2017), it was expected that pesticide mixtures would exert synergistic effects, resulting in greater toxicity than predicted from the effects of individual components.

## Materials and methods

### Chemicals used and experimental design

To assess the influence of plant protection products on the non-target plant species, six active compounds were selected:

- insecticides – pirimicarb (2-(Dimethylamino)−5,6-dimethylpyrimidin-4-yl dimethylcarbamate), belonging to carbamate compounds group and acetamiprid (N-[(6-chloro-3-pyridyl)methyl]-N'-cyano-N-methyl-acetamidine) – representative of neonicotinoid compounds;
- herbicides – glyphosate (N-(Phosphonomethyl)glycine), which is organophosphorus compound and fluroxypyr ([(4-Amino-3,5-dichloro-6-fluoropyridin-2-yl)oxy]acetic acid), from pyridine compounds family;
- fungicides – tebuconazole ((RS)- 1-(4-Chlorophenyl)- 4,4-dimethyl-3-(1H, 1,2,4-triazol-1-ylmethyl)pentan- 3-ol), belonging to triazoles and azoxystrobin (methyl (E)−2-{2-[6-(2-cyanophenoxy)pyrimidin-4-yloxy]phenyl}−3-methoxyacrylate) – strobilurin compound.

These compounds are commonly used in agriculture and belong to a widespread group of toxic compounds. Their diversified mode of action (Table 1) and various physicochemical properties could affect their different behaviour in soil and affect non-target plant species. The studied active substances of pesticides were purchased as analytical standards from Merck (Darmstadt, Germany). Due to their relatively low water solubility (Table 1), except for glyphosate, stock solutions of all pesticides were prepared in analytical-grade acetone of analytical grade. The working standard pesticide solutions used in the proper experimental part were prepared in ultrapure water (Merck Millipore DirectQ3 system, Warsaw, Poland). Eleven experimental variants were tested, including individual pesticides, two-component and three-component mixtures, and a control group. The selected combinations reflect realistic field scenarios in winter wheat (*Triticum aestivum*), which is one of the most widely cultivated and economically important crops in temperate regions, covering large agricultural areas. In this crop, these pesticides are commonly applied either as tank mixes or sequentially during a single growing season to manage multiple pests. The specific application rates and resulting soil concentrations used in the experiments are summarized in Table S1. The treatments were as follows: 1) Pirimicarb, 2) Acetamiprid, 3) Glyphosate, 4) Fluroxypyr, 5) Tebuconazole, 6) Azoxystrobin, 7) Pirimicarb + Glyphosate, 8) Acetamiprid + Tebuconazole, 9) Fluroxypyr + Azoxystrobin, 10) Pirimicarb + Glyphosate + Tebuconazole, 11) Acetamiprid + Fluroxypyr + Azoxystrobin. Ultrapure water served as the control treatment and was also used as the solvent for pesticide mixture applications. Additionally, an acetone control was included in the preliminary study; however, we did not observe any significant differences compared to the standard water control, and therefore it was not included in the main experiment.

**Table 1:** Properties and application details of pesticide active substances used in this study

Pesticide	Chemical Group & Mode of Action	Use	Water Solubility [mg L⁻^1^]	Acetone Solubility (mg L⁻^1^)	K_f_ (mL g⁻^1^)	Log P [pH 7]	pKa	DT50 (Field) [days]
Pirimicarb	Carbamate (Acetylcholinesterase (AChE) inhibitor)	Insecticide	3.10	280,000.00	2.8	1.7	4.4	9
Acetamiprid	Neonicotinoid (Nicotinic Acetylcholine Receptor (nAChR) modulator)	Insecticide	2.95	200,000.00	1.58	0.8	0.7	3
Glyphosate	Organophosphorus (EPSPS enzyme inhibitor in plants)	Herbicide	100,000.00	0.60	54.2	−6.28	2.34	6.45
Fluroxypyr	Pyridine (Synthetic auxin – Growth regulator disruption)	Herbicide	6.50	9200.00	1.20	0.04	2.94	30
Tebuconazole	Triazole (Sterol biosynthesis inhibitor – Fungal cell membrane disruption)	Fungicide	36.00	200.00	12.69	3.7	5.0	47.1
Azoxystrobin	Strobilurin (Mitochondrial respiration inhibitor – QoI)	Fungicide	6.70	86,000.00	7.35	2.5	n/a*	180.7

*n/a*: Not applicable

Mode of action (MOA) classifications were obtained from IRAC (2023), FRAC (2023), and HRAC (2023)

DT50 (Field) – Field half-life of the pesticide, obtained from the Pesticide Properties DataBase (PPDB), University of Hertfordshire, 2024

Water and acetone solubility values were obtained from PPDB, University of Hertfordshire, 2024

Kf – Freundlich sorption coefficient describing the binding strength of the pesticide to soil, obtained from PPDB, University of Hertfordshire, 2024

Pesticides were mixed with soil at concentrations reflecting typical field residue levels based on standard agricultural practices (Table S1). These concentrations were calculated using the PERSAM software (EFSA) (Joris et al., 2023), which estimates pesticide levels based on field application rates of each active ingredient. The reference application rates were taken from pesticide labels for winter wheat. The calculations also took into account soil properties, application type (presowing or post-emergence), and crop cover conditions. For consistency, all pesticides were assumed to be applied under low vegetation conditions (Table S1). The following pesticide concentrations were tested in the soil (mg/kg): tebuconazole (1.688 mg/kg), acetamiprid (0.451 mg/kg), pirimicarb (0.844 mg/kg), glyphosate (9.721 mg/kg), fluroxypyr (1.350 mg/kg), and azoxystrobin (1.688 mg/kg).

The mixtures were prepared by adding the doses corresponding to those used in single applications (Table S1) and dissolving them in the same volume of water.

### Soil used in the study and spiking procedure

The soil material utilised in the study was collected from the topsoil horizon (0–20 cm) of uncultivated Umbrisol [IUSS Working Group WRB, 2015], located in a rural area near Wrocław, Poland (51.171298, 17.163300). Sampling was performed using a stainless-steel soil auger (diameter 5 cm) across an area of approximately 1 hectare. The average sample consisted of 30 subsamples, collected randomly and mixed. The analytical soil material was then air-dried, ground, and sieved (< 2 mm).

The soil used in the experimental part was characterised by a relatively low organic carbon content of 0.53% (Vario Macro Cube CN Elementar Analyser System GmbH, Germany). Its pH measured potentiometrically in a distilled water suspension was equal to 6.81 (soil: water ratio of 1:5 (v/v), Mettler Toledo, Columbus, OH, USA) and bulk density of 1.49 g/cm^3^ measured by Kopecky’s ring method (Spasić et al., 2023). The material represented sandy loam texture (the most common group of agricultural soils that are found in the studied area (Kabała et al., 2015), estimated by sieve and sedimentation method.

Soil treatments with single pesticides and their mixtures were prepared in 1 L polypropylene containers, each containing 780 g of air-dried soil. The soil was spiked with 130 cm^3^ of aqueous working solutions of the tested pesticide(s). In the case of binary mixtures, both active substances were applied simultaneously, each at a concentration corresponding to its recommended field application rate (as specified in Table S1), consistent with the concentrations used in the respective single-compound treatments. The total solution volume in all experimental variants was kept constant (130 cm^3^) to ensure uniform soil moisture conditions across treatments. After the addition of the pesticide solution(s), the soil was allowed to equilibrate for 2 h following initial mixing to ensure uniform distribution of the applied substances. All pesticide solutions were prepared freshly on the day of application to prevent degradation of the active ingredients. Each treatment, including controls, was performed in six replicates to ensure statistical robustness.

### Tests on plants

The effect of individual pesticides in soil or their mixtures on plant germination and growth was estimated using the Phytotoxkit microbiotest. It assesses two effects in test soil compared to control soil: the reduction in seed germination and root growth inhibition, following the principles of ISO standard 11,269–1 (Determination of the effects of pollutants on soil flora – Part 1. Method for the measurement of inhibition of root growth), on measuring pollutant effects on soil flora. Additional endpoints, such as shoot growth and morphological characteristics of early plant stages, can also be analysed. Tests were conducted according to the producer’s instructions with some minor modifications. Three species of test plants were used for the experiment:

- the monocotyl Sorgho (*Sorghum saccharatum*),
- the dicotyl garden cress (*Lepidium sativum*),
- the dicotyl mustard (*Sinapis alba*).

These plants are recommended as test plants by the Phytotoxkit Microbiotest producer due to their relatively high germination capacity and fast growth rate. The Phytotoxkit used unique, flat, and shallow transparent test plates with two compartments. The lower compartment contained 130 g of previously spiked soil (variants described above). After placing the soil in the lower compartment, its surface was levelled and moistened with 5 ml of distilled water. Seeds of the selected test plants were evenly arranged in a straight line along the central part of a black filter paper, which was placed on the soil surface. Ten seeds of each test plant were used per plastic plate. Six replicates were conducted for each plant and soil variant. The test plates were then sealed with a transparent cover using a unique click system, placed vertically in a holder, and incubated in a dark growth chamber at 25 °C (± 1 °C) for 3 days. After this time, photos of the test plates were taken with a digital camera, and the number of germinated seeds on each plastic plate was read (Table S2). Each germinated test plant’s root and stem lengths were measured using the Image J program (National Institutes of Health, USA; https://imagej.nih.gov/ij/).

### Data analysis

Based on the raw data from 6 replications from each treatment, the following ecotoxicological responses were calculated:

$$\text{Germination inhibition }= \frac{N \times 100}{K} - 100$$

where:

N—number of germinated seeds in the test soil,

K—number of germinated seeds in the control soil.

$$\text{Root growth inhibition }= \frac{RR \times 100}{RK} - 100$$

where:

RR—root length in test soil.

RK—root length in control soil

$$\text{Stem growth inhibition }=\frac{SR \times 100}{SK} - 100$$

where:

SR—stem length in test soil.

SK—stem length in control soil.

The normality of the calculated responses was tested using the Shapiro–Wilk test. The effects of the toxicity of single substances (each expressed as response in comparison to control) were analysed using one-way analysis of variance (ANOVA). Subsequently, the effects of the mixtures were compared to those of the individual components, also using one-way ANOVA. Post-hoc comparisons were performed using Tukey’s HSD test to identify statistically significant differences between groups. Only the significant effects were further shown on the graphs.

The toxicity of pesticide mixtures was evaluated only when the combined application produced stronger effects than individual substances, as determined by ANOVA. Three predictive models—additive, dominance, and multiplicative—were applied to assess mixture effects using the responses to single pesticides as a basis for prediction. The models were previously used in the studies of Meidl et al., (2024) and has been previously descibed by Rodney et al. (2013). In these models, *E*_*i*_ represents the effect of an individual component iii in the mixture. In the additive model, the total effect of the mixture is assumed to equal the sum of the effects of the individual components, implying the absence of synergistic or antagonistic interactions.

$${\text{E}}_{add}={\sum }_{i}{E}_{i}$$

The dominance model assumes that the component with the strongest individual effect determines the overall toxicity, potentially obscuring interactive effects such as synergism or antagonism.

$${\text{E}}_{dom}=\text{max}({E}_{i})$$

The multiplicative model assumes independent action of the components, where synergism is inferred if the observed combined effect exceeds the predicted one.

$${\text{E}}_{mult}=1-\uppi (1-{E}_{i})$$

To quantify how well each model predicted the observed outcomes, the Taylor method (Taylor, 2001) was used, incorporating three statistical metrics: the Pearson correlation coefficient (CC), root-mean-square error (RMSE), and standard deviation (SD). These metrics allow a visual and quantitative assessment of model performance: higher CC values indicate stronger agreement between observed and predicted values, while lower RMSE and SD values indicate better fit and accurate representation of data variability. In this study, models showing relatively high correlation and low RMSE, with SD values similar to the observed data, were considered to provide the best fit. Statistical analyses were performed using Origin Pro and R Studio Version 1.6.0.

## Results

### Effects of single substances

**Fig. 1:**
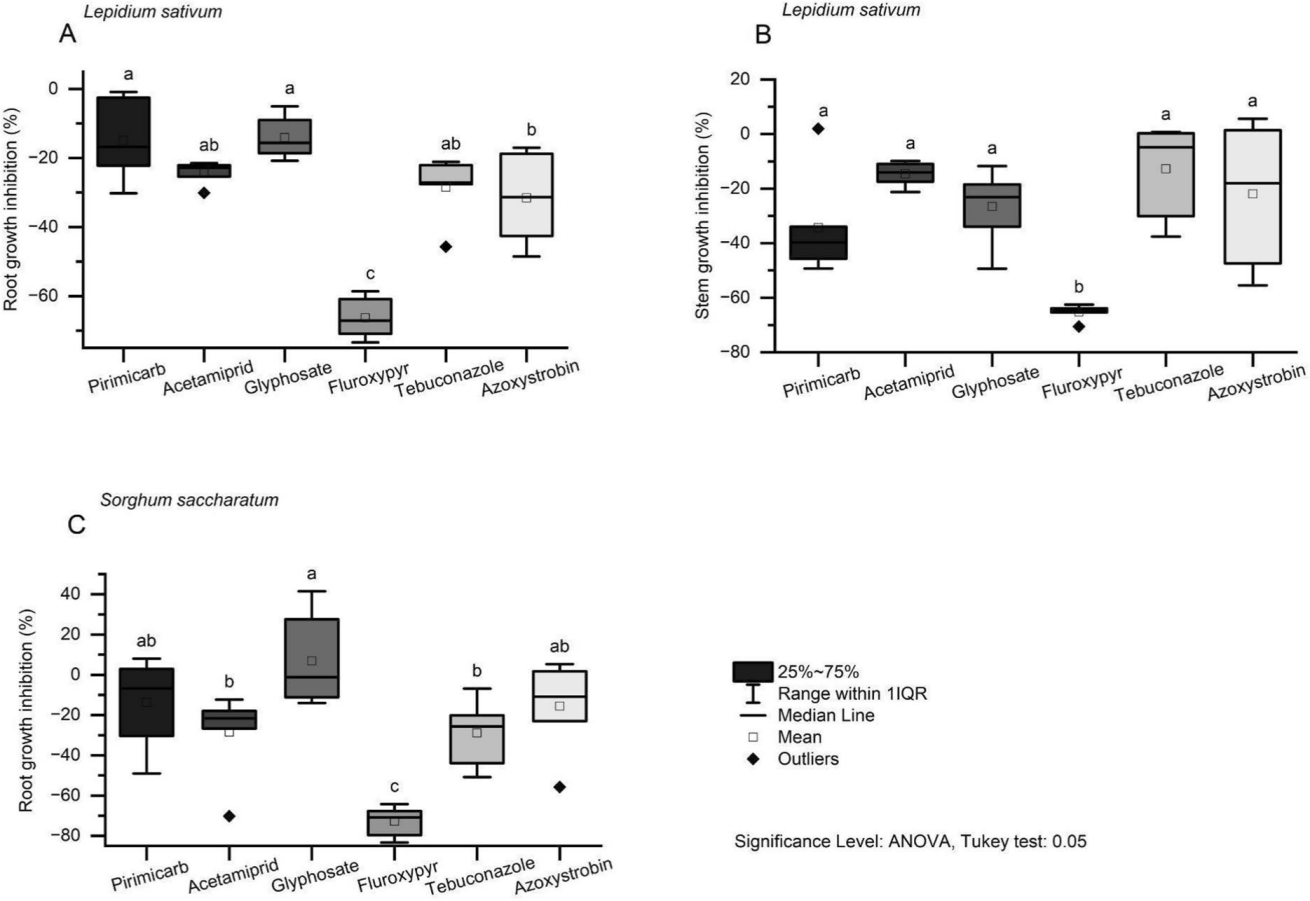
The effects of single pesticide use on the root growth inhibition of *Lepidium sativum* (**A**) and *Sorghum saccharatum* (**C**)*,* as well as stem growth inhibition of *Lepidium sativum* (**B**). Note: Different lowercase letters indicate statistically significant differences between treatments (ANOVA., Tukey’s test)

For both *Lepidium sativum* and *Sorghum saccharatum*, the six tested substances exhibited varying degrees of phytotoxicity (Fig. 1, Table S3). Among them, fluroxypyr consistently demonstrated the highest toxicity, causing the greatest inhibition of root and stem growth in *L. sativum* (Fig. 1A–B) and root growth in *S. saccharatum* (Fig. 1C). In *L. sativum*, azoxystrobin also significantly inhibited root growth (Fig. 1A), while fluroxypyr caused the most pronounced suppression of stem growth (Fig. 1B). For *S. saccharatum*, both tebuconazole and acetamiprid resulted in a significant reduction in root growth compared to the other treatments (Fig. 1C).

### Effects of pesticide mixtures

**Fig. 2:**
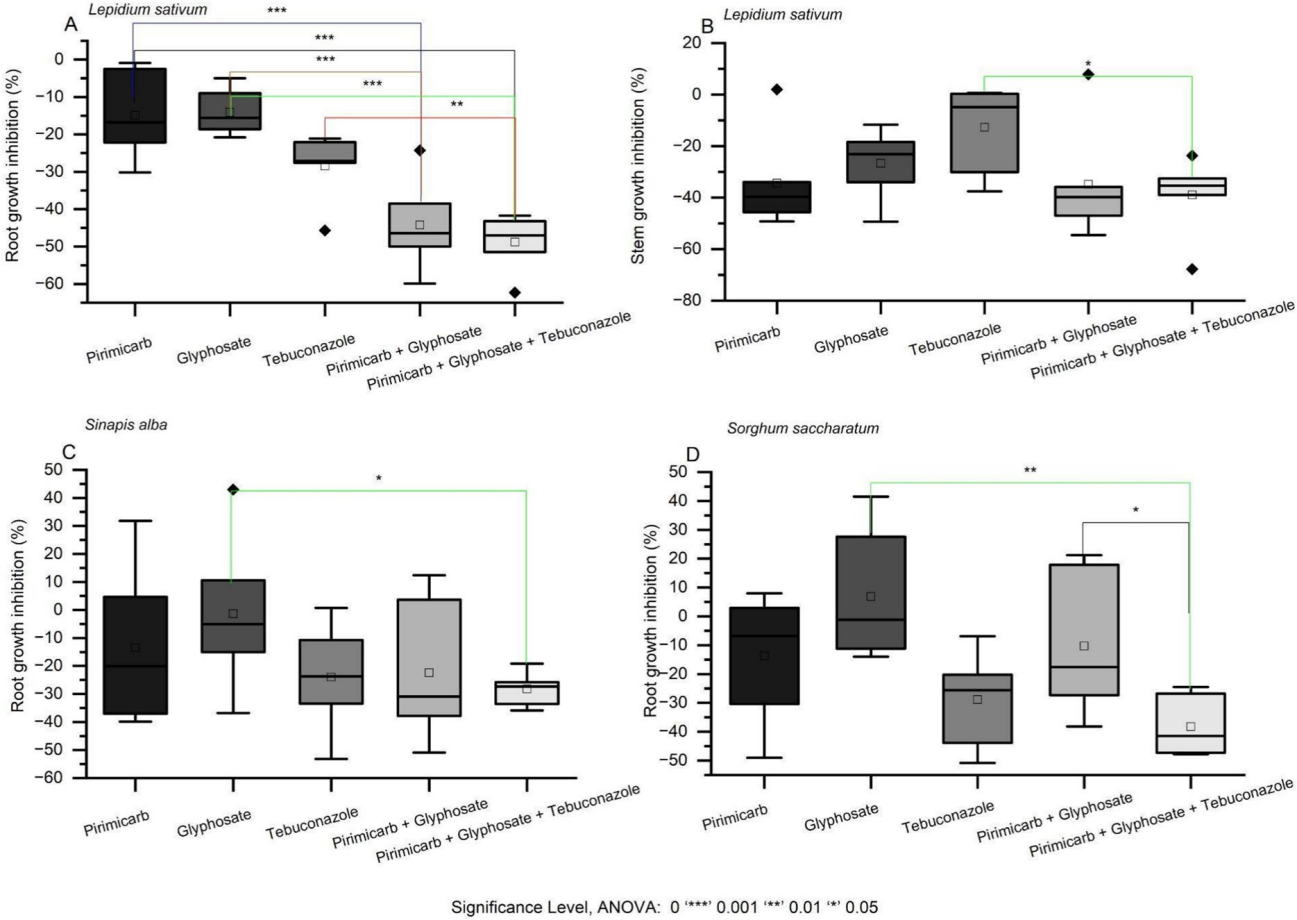
The toxicity effects of pesticide mixtures of pirimicarb, glyphosate, and tebuconazole on plant growth responses. Note: Brackets indicate statistically significant differences between each single substance and the corresponding mixture, as determined by the ANOVA

The toxicity of pesticide mixtures was evaluated on *Lepidium sativum*, *Sinapis alba*, and *Sorghum saccharatum*, focusing on both root and stem growth inhibition. Each mixture was assessed not only for its overall phytotoxic effect compared to individual compounds (Fig. 2., Table S4), but also using predictive models—additive, multiplicative, and dominance—to infer the likely interactions driving mixture toxicity (Table 2).

**Table 2:** The comparisons of predicted models of pesticide toxicity responses additive, multiplicative and dominance) to the data obtained

Pant response	Model	Mean	Standard Deviation	Variance	Centered RMS Difference	Correlation Coefficient
	Pirimicarb + Glyphosate + Tebuconazole					
*Lepidium sativum*—root growth inhibition						
	Additive	0.57	0.18	0.03	0.15	0.32
	Multiplicative	0.01	0.00	0.00	0.05	0.25
	Dominance	0.28	0.09	0.01	0.09	0.24
*Lepidium sativum*—stem growth inhibition						
	Additive	0.74	0.34	0.11	0.37	0.07
	Multiplicative	0.01	0.02	0.00	0.22	0.41
	Dominance	0.39	0.14	0.02	0.28	−0.13
*Sinapis alba*—stem growth inhibition						
	Additive	0.11	0.47	0.22	2,281,002.00	−0.08
	Multiplicative	0.00	0.06	0.00	2,276,432.00	0.14
	Dominance	0.40	0.15	0.02	2,285,914.00	−0.62
*Sorhhum saccharatum*—root growth inhibition						
	Additive	0.36	0.33	0.11	0.29	0.24
	Multiplicative	−0.01	0.02	0.00	0.10	−0.34
	Dominance	0.34	0.18	0.03	0.17	0.15
Pirimicarb + Glyphosate						
*Lepidium sativum*—root growth inhibition						
	Additive	0.29	0.13	0.02	0.29	−0.31
	Multiplicative	0.02	0.01	0.00	0.23	−0.47
	Dominance	0.19	0.08	0.01	0.24	−0.02
Fluroxypyr + Azoxystrobin						
*Lepidium sativum*—root growth inhibition						
	Additive	0.98	0.15	0.02	0.08	0.89
	Multiplicative	0.21	0.09	0.01	0.04	0.87
	Dominance	0.66	0.06	0.00	0.07	0.36
*Lepidium sativum*—stem growth inhibition						
	Additive	0.87	0.24	0.06	0.19	0.69
	Multiplicative	0.14	0.16	0.03	0.12	0.65
	Dominance	0.65	0.03	0.00	0.06	0.17
*Sinapis alba*—root growth inhibition						
	Additive	0.38	0.24	0.06	0.25	−0.20
	Multiplicative	0.02	0.06	0.00	0.10	−0.28
	Dominance	0.34	0.16	0.03	0.16	−0.01
*Sinapis alba*—stem growth inhibition						
	Additive	0.61	0.14	0.02	0.14	−0.01
	Multiplicative	0.05	0.09	0.01	0.11	−0.11
	Dominance	0.47	0.11	0.01	0.11	0.10
*Sorhhum saccharatum*—root growth inhibition						
	Additive	0.88	0.25	0.06	0.28	−0.36
	Multiplicative	0.12	0.16	0.03	0.20	−0.20
	Dominance	0.73	0.07	0.01	0.16	−0.67
*Sorhhum saccharatum* -stem growth inhibition						
	Additive	0.45	0.26	0.07	0.33	−0.67
	Multiplicative	0.02	0.10	0.01	0.18	−0.27
	Dominance	0.42	0.15	0.02	0.25	−0.79
Acetamiprid + Fluroxypyr + Azoxystrobin						
*Lepidium sativum*—root growth inhibition						
	Additive	122,033.00	0.12	0.01	0.12	−0.32
	Multiplicative	0.05	0.02	0.00	0.04	−0.40
	Dominance	0.66	0.06	0.00	0.05	0.37
*Lepidium sativum*—stem growth inhibition						
	Additive	101,788.00	0.28	0.08	0.25	0.24
	Multiplicative	0.03	0.03	0.00	0.07	0.33
	Dominance	0.65	0.03	0.00	0.07	0.31
*Sinapis alba*—root growth inhibition						
	Additive	0.62	0.29	0.08	0.27	−0.20
	Multiplicative	0.00	0.02	0.00	0.02	0.84
	Dominance	0.37	0.11	0.01	0.13	−0.69
*Sinapis alba*—stem growth inhibition						
	Additive	0.58	0.22	0.05	0.24	−0.37
	Multiplicative	−0.01	0.03	0.00	0.08	−0.13
	Dominance	0.47	0.11	0.01	0.16	−0.89
*Sorhhum saccharatum*—root growth inhibition						
	Additive	116,733.00	0.22	0.05	0.21	−0.17
	Multiplicative	0.02	0.03	0.00	0.04	0.30
	Dominance	0.73	0.07	0.01	0.07	0.16
*Sorhhum saccharatum* -stem growth inhibition						
	Additive	0.59	0.32	0.10	0.23	0.66
	Multiplicative	0.00	0.02	0.00	0.10	0.42
	Dominance	0.47	0.15	0.02	0.10	0.70
Acetamiprid + Tebuconazole						
*Lepidium sativum*—root growth inhibition						
	Additive	0.27	0.19	0.04	0.37	−0.71
	Multiplicative	0.02	0.03	0.00	0.24	−0.66
	Dominance	0.21	0.11	0.01	0.31	−0.78
*Lepidium sativum*—stem growth inhibition						
	Additive	0.53	0.10	0.01	0.15	−0.62
	Multiplicative	0.07	0.02	0.00	0.10	−0.59
	Dominance	0.29	0.09	0.01	0.15	−0.76
*Sinapis alba*—root growth inhibition						
	Additive	0.48	0.06	0.00	0.11	−0.31
	Multiplicative	0.03	0.04	0.00	0.09	−0.16
	Dominance	0.36	0.11	0.01	0.12	0.09
*Sorhhum saccharatum* -stem growth inhibition						
	Additive	0.03	0.39	0.15	0.51	−0.50
	Multiplicative	−0.02	0.06	0.00	0.21	0.38
	Dominance	0.23	0.25	0.06	0.38	−0.44

Mean: average predicted effect of the mixture, Standard Deviation (SD): spread of predicted values around the mean,Variance: square of the SD, indicating variability of predictions, Centered Root-Mean-Square Difference (RMSD): overall deviation between predicted and observed effects; lower values indicate better accuracy, Correlation Coefficient (CC): Pearson correlation between predicted and observed values; values close to 1 indicate strong agreement, while negative values indicate poor or inverse prediction

Models showing relatively high CC, low RMSD, and SD similar to observed data are considered to provide the best fit

The mixture of pirimicarb, glyphosate, and tebuconazole caused significantly higher inhibition of *L. sativum* root growth compared to any single compound and increased stem inhibition relative to tebuconazole alone (Fig. 2A–B, Table S4). In *S. alba*, root growth was more inhibited than by glyphosate used as a single substance (Fig. 2C). In contrast, in *S. saccharatum*, the three-component mixture exceeded the toxicity of both glyphosate alone and the binary combination of pirimicarb and tebuconazole (Fig. 2D). Predictive modeling indicated that the additive model most accurately described these effects (Table 2). For *L. sativum* root growth, the additive model yielded a CRMSD of 0.15 and a correlation coefficient (CC) of 0.32, while for stem growth, the additive model showed a CRMSD of 0.37 and a CC of 0.07 (Table 2). In *S. alba* and *S. saccharatum*, the additive model similarly provided the closest fit compared to multiplicative and dominance models, which tended to underestimate or overestimate inhibition.

**Fig. 3:**
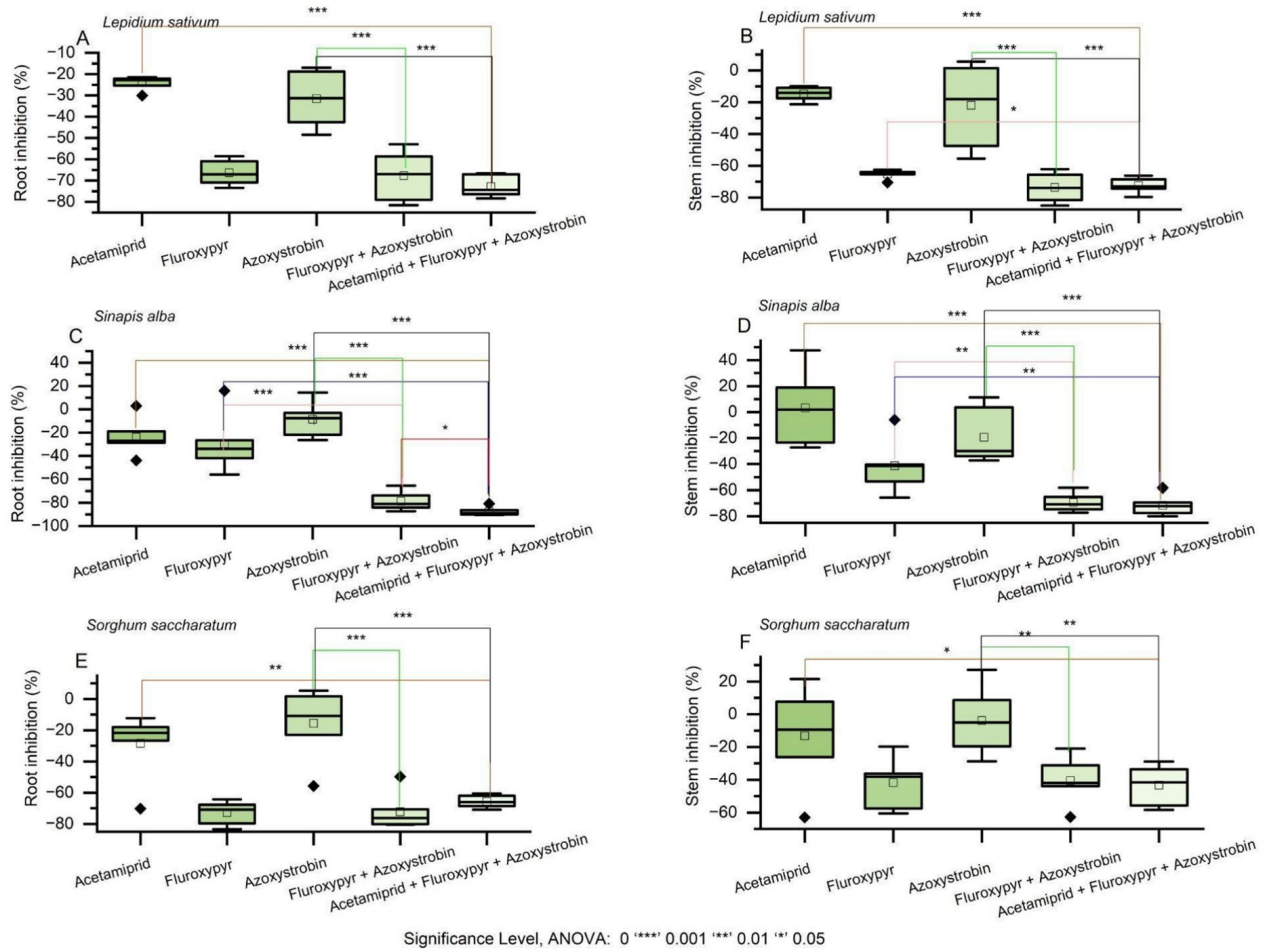
The toxicity effects of pesticide mixtures of acetamiprid, fluroxypyr and azoxystrobin on plant growth responses. Note: Brackets indicate statistically significant differences between each single substance and the corresponding mixture, as determined by the ANOVA

The mixture of acetamiprid, fluroxypyr, and azoxystrobin exhibited increased toxicity across all tested species in comparison to single substances. For *L. sativum*, root inhibition was significantly higher than any single compound or two-component mixture (Fig. 3A, Table S4), with the additive model providing the closest predictive fit (CRMSD = 0.12,

CC = –0.32). Stem growth inhibition was also greater under the mixture, with additive model values of CRMSD = 0.25 and CC = 0.24 (Fig. 3B). In *S. alba*, root and stem growth inhibition were substantially increased (Figs. 3C–D), and the additive model again offered the best description (CRMSD = 0.27, CC = –0.20; stem: mean = 0.58, CRMSD = 0.24, CC = –0.37). For *S. saccharatum*, both root and stem inhibition were higher than under any single or binary combination (Figs. 3E–F), with additive predictions of root: CRMSD = 0.21, CC = –0.17, and stem: mean = 0.59, CRMSD = 0.23, CC = 0.66.

**Fig. 4:**
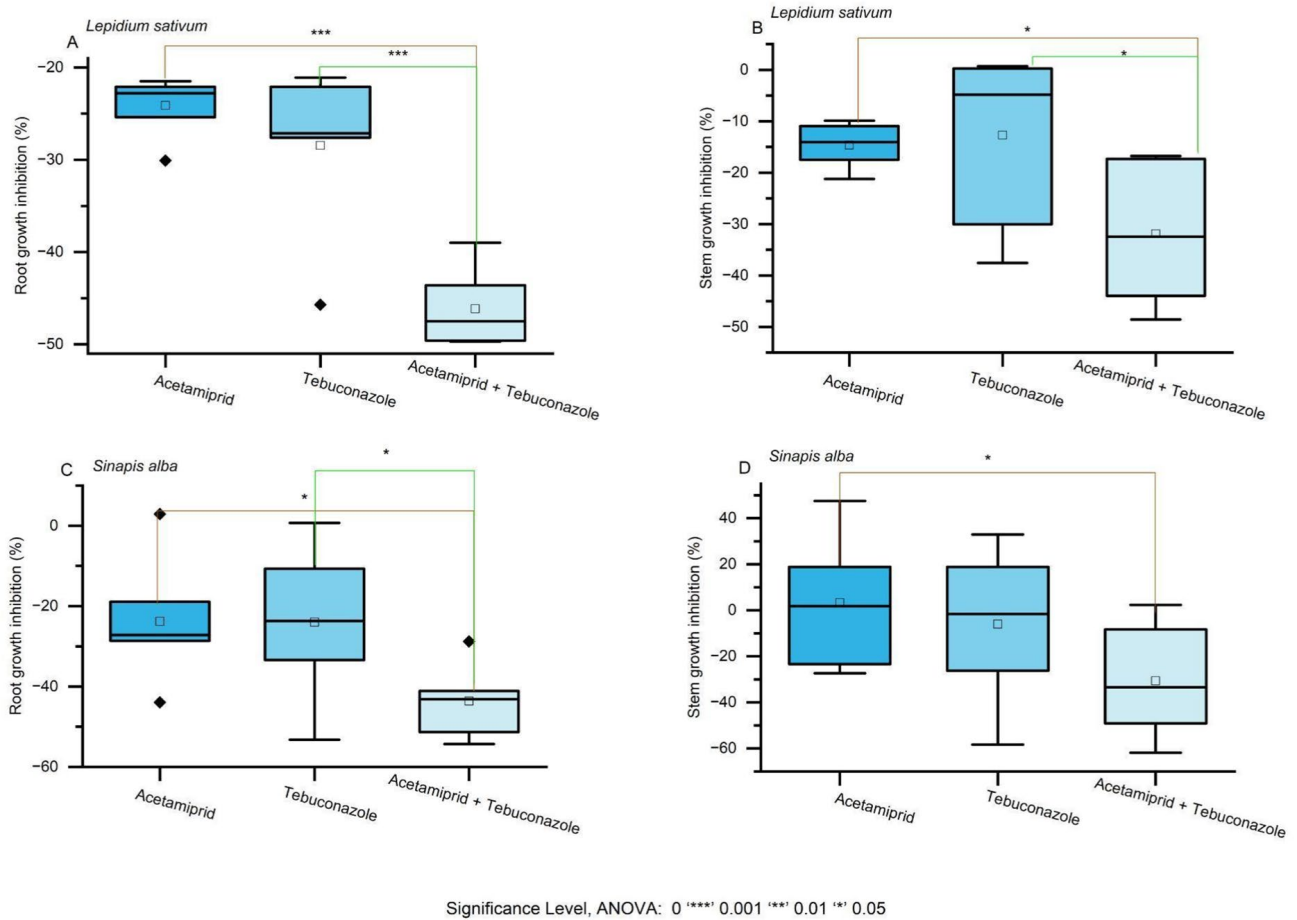
The toxicity effects of pesticide mixtures of acetamiprid and tebuconazole on plant growth responses. Note: Brackets indicate statistically significant differences between each single substance and the corresponding mixture, as determined by the ANOVA

The combination of acetamiprid and tebuconazole enhanced toxicity relative to individual components (Fig. 4, Table 2). In *L.sativum*, root growth inhibition exceeded that caused by acetamiprid or tebuconazole alone (Fig. 4A, Table S4), with the additive model predicting a mean inhibition of 0.27, CRMSD = 0.37, and CC = –0.71 (Table 2). Stem inhibition followed a similar pattern, with additive model predictions of mean = 0.53, CRMSD = 0.15, and CC = –0.62 (Fig. 4B, Table 2). In *S. alba*, root inhibition was increased by the mixture, with the additive model predicting mean = 0.48, CRMSD = 0.11, and CC = –0.31 (Fig. 4C Table 2). For *S. saccharatum*, stem inhibition was more pronounced than with single treatments, with the additive model predicting mean = 0.03, CRMSD = 0.51, and CC = –0.50 (Fig. 4D, Table 2).

## Discussion

This is the first study assessing the impact of combined pesticide use on non-target plant species, highlighting the unintended side effects of pesticide application under semi-realistic environmental exposure conditions. Most existing studies focus on the toxicity of individual active substances in laboratory environments; in contrast, our approach considers multiple pesticides and their interactions in a more environmentally relevant context.

Our indicate that fluroxypyr exerts toxic effects on non-target plants, partially supporting the hypothesis that herbicides tend to be more harmful to plants, due to its specific mode of action. Fluroxypyr, designed to target broadleaf weeds, negatively affected all three studied plant species—*Lepidium sativum, Sorghum saccharatum, and Sinapis alba* (HRAC (2023). In contrast, glyphosate, which disrupts dicotyledonous and monocotyledonous weeds (HRAC, 2023), showed no observable toxic effects. The distinct mechanisms of these herbicides explain this difference: fluroxypyr mimics plant hormones, causing uncontrolled growth and eventual death, while glyphosate inhibits an enzyme crucial for amino acid synthesis (Duke, 2021). Fluroxypyr, used in grasslands to control invasive plant species, has been shown to directly impact native plant species or indirectly affect them through increased competition with exotic species (Smith et al., 2023). For glyphosate, instead, slight non-target plant (mostly soybean) responses or no toxicity effects are described in the review of Olszyk et al. (2004), similar to our findings. In contrast, studies of other authors (Ferreira et al., 2023; Florencia et al., 2017) on seedlings in greenhouse conditions proved that glyphosate exerted distinct sublethal toxicity to all studied plant species (Asteraceae, Poaceae, Bignoniaceae, Solanaceae, Convolvulaceae) at the field application rates. However, what has to be taken into consideration, in the study above, herbicides were applied directly on plants and were not mixed with the soil, as in our experiment (Ferreira et al., 2023). Our study was carried out under controlled conditions, focusing exclusively on the effects observed in the seedling stage. Previous research indicates that seedlings tend to be more susceptible to pesticide exposure than plants at later developmental stages (Zwerger & Pestemer, 2000). Moreover, studies have shown that sublethal impacts of herbicide treatment—such as reduced biomass or seed output—may become evident only at more advanced stages of plant development (Carpenter & Boutin, 2010; Strandberg et al. 2017). Additionally, this study identified slight toxic effects of azoxystrobin on *Lepidium sativum*, and of acetamiprid and tebuconazole on *Sorghum saccharatum*. The phytotoxicity of azoxystrobin has previously been confirmed in laboratory experiments on *Lepidium sativum* and *Sinapis alba*, even at lower concentrations (0.110 mg/kg) than the dose applied in the present study (1.688 mg/kg) (Baćmaga et al., 2024). Tebuconazole has also been shown to suppress seed germination in mustard (Saneeva et al., 2022). In contrast, there is currently no published data on the effects of acetamiprid on plant growth. Also, the persistence of pesticides in the soil may influence their toxic effects (Van Hall et al., 2023). Among the tested substances, azoxystrobin (DT₅₀ = 181 days), tebuconazole (DT₅₀ = 47.1 days) and fluroxypyr (DT₅₀ = 47.1 days) exhibited the most prolonged soil half-lives. In contrast, the DT₅₀ values for the other pesticides ranged from 3 to 9 days (Table 1, PPDB (2023)).

Several recent reports indicate that pesticide residues, including herbicides, frequently coexist in soils with other active ingredients (Kalyabina et al., 2021; Silva et al., 2023). Such co-occurrence often results from the widespread agricultural practice of tank mixing, as seen in crops like corn, cotton, and wheat (Daramola et al., 2023). Previous studies have shown that mixtures can produce synergistic, antagonistic, or additive effects, depending on the compounds and concentrations involved. Herbicide mixture effects on plants and algae varied depending on the species and test systems, with approximately 70% of mixtures showing antagonistic effects (Cedergreen et al., 2007). For example, combinations of glyphosate and dicamba have exhibited antagonistic effects on native plants and non-genetically engineered crops, with certain species being more sensitive than others (Olszyk et al., 2015). Even at low concentrations, pesticide mixtures can inhibit root elongation, increase aberrant cell frequency, and cause cell death in plants (Miranda et al., 2023). Glyphosate-based herbicides, in particular, have been found to delay seed germination and reduce shoot biomass in crops such as faba bean, oat, and turnip rape (Helander et al., 2019). The persistence of these compounds in soil can lead to long-term impacts on non-target plants, potentially altering plant community structure and ecosystem dynamics (Karthikeyan et al., 2004).

In the present study, we hypothesised that pesticide mixtures would exhibit synergistic effects, producing greater toxicity than predicted from the effects of individual components alone. This hypothesis was supported for all tested two-component combinations (fluroxypyr + azoxystrobin, pirimicarb + glyphosate, acetamiprid + tebuconazole) and three-component combinations (acetamiprid + fluroxypyr + azoxystrobin, pirimicarb + glyphosate + tebuconazole), where combined toxicity exceeded that of the single active ingredients. However, the addition of a third compound significantly increased toxicity in only a few cases, such as when tebuconazole was added to the pirimicarb–glyphosate mixture. Overall, plant responses were best explained by the additive model, which assumes that the total effect of a mixture equals the sum of the individual effects, indicating no consistent synergistic or antagonistic interactions (Schäfer & Piggott, 2018).

This predominance of additive responses is consistent with evidence from other biological systems. In honey bees, pesticide mixture effects can be reliably predicted by the Concentration Addition model (Taenzler et al., 2023), and in aquatic organisms, repeated and multiple pesticide exposures typically result in additive toxicity, with only occasional cases of greater-than-additive responses (Dennis et al., 2012). Nonetheless, the documented instances of both synergistic and antagonistic interactions—particularly involving glyphosate-based herbicides and dicamba—highlight the complexity of mixture effects and the potential risks to non-target plant species. These findings underscore the need for further research to better characterise the mechanisms and environmental relevance of pesticide mixture interactions, ultimately informing more effective risk assessment and management strategies for agricultural and natural ecosystems.

## Conclusions

This study demonstrated that pesticide mixtures can pose greater risks to non-target plant species than individual active substances applied alone. Among the tested compounds, fluroxypyr showed the highest phytotoxicity across all species, while glyphosate had no measurable effects under the conditions used. The combination of pesticides often increased toxicity, with several two- and three-component mixtures exhibiting stronger effects than expected based on individual components. Notably, even mixtures without herbicides, such as acetamiprid with tebuconazole, caused unexpected toxicity. The additive model most consistently explained plant responses to pesticide mixtures, suggesting that, in many cases, combined effects can be predicted from the sum of individual toxicities. However, some mixtures showed signs of synergism, particularly when one component was notably more toxic, which was better captured by the dominance model. The multiplicative model had limited predictive value. These results highlight the ecological relevance of mixture toxicity and the need to consider interactive effects in pesticide risk assessments, especially for early plant development stages, taking into account the temporal degradation aspect.

## Supplementary Information

Below is the link to the electronic supplementary material.

Supplementary file 1 (DOCX 39.1 KB)

## Data Availability

No datasets were generated or analysed during the current study.
