# Supplementary material for: Assessing the ecotoxicological effects of pesticides on non-target plant species
Source: Environ Monit Assess. 2025 Aug 27;197(9):1047. doi: 10.1007/s10661-025-14532-2 (PMC12380957; doi:10.1007/s10661-025-14532-2)
Supplement: Supplementary file 1 — (DOCX 39.1 KB) [file 10661_2025_14532_MOESM1_ESM.docx]

**Table S1 Input data used in the PERSAM software and concentration of pesticides tested**

| **Substance** | **Rate (g/ha)** | **Total Soil Concentration (mg/kg)** |
| --- | --- | --- |
| Tebuconazole | 250 | 1.688 |
| Acetamiprid | 60 | 0.451 |
| Pirimicarb | 125 | 0.844 |
| Glyphosate | 1440 | 9.721 |
| Fluroxypyr | 200 | 1.350 |
| Azoxystrobin | 250 | 1.688 |
| **Experimental conditions:**  Time since first application: 0 days - assumed parameter  Growth stage: BBCH 0–9 (Annual crop) - assumed parameter  Soil layer: 0–5 cm - assumed parameter  Temperature: 20°C - assumed parameter  Organic carbon: 0.56% - measured parameter  Bulk density: 1.49 g/cm³ - measured parameter | | |

**Table S2 Mean germination inhibition (%) of seeds of three test plants under the influence of individual substances or their mixtures**

| **Treatment** | ***Lepidium sativum*** | ***Sinapis alba*** | ***Sorghum saccharatum*** |
| --- | --- | --- | --- |
| Acetamiprid | 0.0 | -6.5 | -4.7 |
| Pirimicarb | -3.0 | 0.0 | 0.0 |
| Glyphosate | -3.0 | 4.3 | 7.0 |
| Fluroxypyr | 0.0 | -13 | -7.0 |
| Tebuconazole | 0.0 | -10.9 | -11.6 |
| Azoxystrobin | 0.0 | -2.2 | -14.0 |
| Pirimicarb + Glyphosate | 0.0 | -2.2 | 7.0 |
| Acetamiprid + Tebuconazole | -2.0 | 2.2 | -14.0 |
| Fluroxypyr + Azoxystrobin | -10 | -6.5 | 2.3 |
| Pirimicarb + Glyphosate + Tebuconazole | -2.0 | -2.2 | -2.3 |
| Acetamiprid + Fluroxypyr + Azoxystrobin | -8.0 | -6.5 | 18.6 |

**Table S3. Summary of statistical results (ANOVA) of toxicity responses of plants to contaminants to single pesticides substances**

| Response variable | **DF** | **Sum of Squares** | **Mean Square** | **F Value** | **Prob>F** |
| --- | --- | --- | --- | --- | --- |
| *Lepidium sativum -* Root growth inhibition | *5* | 11042.2 | 2208.4 | 29.44129 | <0.0001 |
| Lepidum sativum - Stem growth inhibition | 5 | 11191.45 | 2238.3 | 9.08758 | <0.0001 |
| *Sinapis alba* - Root growth inhibition | 5 | 3455 | 691 | 1.43365 | 0,24 |
| *Sinapis alba* - Stem growth inhibition | 5 | 10751.7 | 2150.3 | 1.94043 | 0,123 |
| *Sorghum saccharatum* - Root growth inhibition | 5 | 21258.9 | 4251.98 | 11.39362 | <0.0001 |
| *Sorghum saccharatum* - Root growth inhibition | 5 | 9461.43 | 1892.47 | 2.23895 | 0,08 |

**Table S4 Summary of statistical results (ANOVA) of toxicity responses of plants to contaminants in pesticide mixtures in comparison to single substances**

| **Group 1** | | **Group 2** | **DF** | **Sum of Squares** | **Mean Square** | **F Value** | **Prob>F** |
| --- | --- | --- | --- | --- | --- | --- | --- |
| ***Lepidium sativum - Root growth inhibition*** | | | | | | | |
| Pirimicarb | | Pirimicarb + Glyphosate | 1 | 2587.2 | 2587.2 | 18.82 | 0.001 |
| Pirimicarb | | Pirimicarb + Glyphosate + Tebuconazole | 1 | 3448 | 3448 | 36.95 | 0.0001 |
| Glyphosate | | Pirimicarb + Glyphosate | 1 | 20029.36 | 20029.36 | 210.65 | <0.0001 |
| Glyphosate | | Pirimicarb + Glyphosate + Tebuconazole | 1 | 3612 | 3612 | 78.02 | <0.0001 |
| Pirimicarb + Glyphosate + Tebuconazole | | Pirimicarb + Glyphosate | 1 | 61.7 | 61.65 | 0.618 | 0.45 |
| Tebuconazole | | Pirimicarb + Glyphosate + Tebuconazole | 1 | 1240.3 | 1240.3 | 18.4 | 0.00159 |
| Acetamiprid | | Acetamiprid + Fluroxypyr + Azoxystrobin | 1 | 7130 | 7130 | 410.2 | <0.0001 |
| Fluroxypyr | | Fluroxypyr + Azoxystrobin | 1 | 5.5 | 5.47 | 0.062 | 0.809 |
| Fluroxypyr | | Acetamiprid + Fluroxypyr + Azoxystrobin | 1 | 128.1 | 128.05 | 4.526 | 0.0593 |
| Azoxystrobin | | Fluroxypyr + Azoxystrobin | 1 | 3910 | 3910 | 25.59 | 0.00049 |
| Azoxystrobin | | Acetamiprid + Fluroxypyr + Azoxystrobin | 1 | 5113 | 5113 | 55.45 | <0.0001 |
| Fluroxypyr + Azoxystrobin | | Acetamiprid + Fluroxypyr + Azoxystrobin | 1 | 80.6 | 80.6 | 0.95 | 0.35 |
| Acetamiprid | | Acetamiprid + Tebuconazole | 1 | 1456.4 | 1456.4 | 105.4 | <0.0001 |
| Tenuconazole | | Acetamiprid + Tebuconazole | 1 | 938.1 | 938.1 | 19.47 | 0.0013 |
| ***Lepidum sativum - Stem growth inhibition*** | | | | | | | |
| Pirimicarb | Pirimicarb + Glyphosate | | 1 | 0.73 | 0.72521 | 0.00175 | 0.97 |
| Pirimicarb | Pirimicarb + Glyphosate + Tebuconazole | | 1 | 63.3 | 63.34 | 0.22 | 0.65 |
| Glyphosate | Pirimicarb + Glyphosate | | 1 | 1292.52 | 1292.52 | 4.97471 | 0.05 |
| Glyphosate | Pirimicarb + Glyphosate + Tebuconazole | | 1 | 456.6 | 456.6 | 2.259 | 0.16 |
| Pirimicarb + Glyphosate + Tebuconazole | Pirimicarb + Glyphosate | | 1 | 51 | 50.5 | 0.142 | 0.71 |
| Tebuconazole | Pirimicarb + Glyphosate + Tebuconazole | | 1 | 2065 | 2064.8 | 8.044 | 0.018 |
| Acetamiprid | Acetamiprid + Fluroxypyr + Azoxystrobin | | 1 | 10032 | 10032 | 457.7 | <0.0001 |
| Fluroxypyr | Fluroxypyr + Azoxystrobin | | 1 | 215.1 | 215.05 | 4.881 | 0.052 |
| Fluroxypyr | Acetamiprid + Fluroxypyr + Azoxystrobin | | 1 | 157.9 | 157.91 | 10.37 | 0.0092 |
| Azoxystrobin | Fluroxypyr + Azoxystrobin | | 1 | 8018 | 8018 | 22.36 | 0.00081 |
| Azoxystrobin | Acetamiprid + Fluroxypyr + Azoxystrobin | | 1 | 7646 | 7646 | 23.19 | 0.00071 |
| Fluroxypyr + Azoxystrobin | Acetamiprid + Fluroxypyr + Azoxystrobin | | 1 | 4.4 | 4.4 | 0.085 | 0.77 |
| Acetamiprid | Acetamiprid + Tebuconazole | | 1 | 897.4 | 897.4 | 8.29 | 0.016 |
| Tenuconazole | Acetamiprid + Tebuconazole | | 1 | 1105 | 1105 | 4.586 | 0.050 |
| ***Sinapis alba - Root growth inhibition*** | | | | | | | |
| Pirimicarb | Pirimicarb + Glyphosate | | 1 | 243 | 243 | 0.35 | 0.57 |
| Pirimicarb | Pirimicarb + Glyphosate + Tebuconazole | | 1 | 656 | 655.6 | 1.61 | 0.23 |
| Glyphosate | Pirimicarb + Glyphosate | | 1 | 1327.2 | 1327.20 | 1.97149 | 0.19 |
| Glyphosate | Pirimicarb + Glyphosate + Tebuconazole | | 1 | 2157 | 4.1 | 5.69 | 0.034 |
| Pirimicarb + Glyphosate + Tebuconazole | Pirimicarb + Glyphosate | | 1 | 100 | 100.3 | 0.304 | 0.594 |
| Tebuconazole | Pirimicarb + Glyphosate + Tebuconazole | | 1 | 52.9 | 52.92 | 0.277 | 0.61 |
| Acetamiprid | Acetamiprid + Fluroxypyr + Azoxystrobin | | 1 | 12218 | 12218 | 97.49 | <0.0001 |
| Fluroxypyr | Fluroxypyr + Azoxystrobin | | 1 | 7321 | 7321 | 22.16 | 0.00083 |
| Fluroxypyr | Acetamiprid + Fluroxypyr + Azoxystrobin | | 1 | 10156 | 10156 | 33.22 | 0.00018 |
| Azoxystrobin | Fluroxypyr + Azoxystrobin | | 1 | 14763 | 14763 | 107.7 | <0.0001 |
| Azoxystrobin | Acetamiprid + Fluroxypyr + Azoxystrobin | | 1 | 18691 | 18691 | 166.4 | <0.0001 |
| Fluroxypyr + Azoxystrobin | Acetamiprid + Fluroxypyr + Azoxystrobin | | 1 | 231.4 | 231.44 | 6.133 | 0.0327 |
| Acetamiprid | Acetamiprid + Tebuconazole | | 1 | 1178 | 1178.1 | 7.393 | 0.0216 |
| Tenuconazole | Acetamiprid + Tebuconazole | | 1 | 1156 | 1156.4 | 5.42 | 0.0422 |
| ***Sinapis alba - Stem growth inhibition*** | | | | | | | |
| Pirimicarb | Pirimicarb + Glyphosate | | 1 | 432 | 432 | 0.42 | 0.53 |
| Pirimicarb | Pirimicarb + Glyphosate + Tebuconazole | | 1 | 2561 | 2560.8 | 4.18 | 0.068 |
| Glyphosate | Pirimicarb + Glyphosate | | 1 | 894.41 | 894.41 | 0.39 | 0.55 |
| Glyphosate | Pirimicarb + Glyphosate + Tebuconazole | | 1 | 0 | 0 | 0 | 1.00 |
| Pirimicarb + Glyphosate + Tebuconazole | Pirimicarb + Glyphosate | | 1 | 889 | 889.2 | 0.866 | 0.37 |
| Tebuconazole | Pirimicarb + Glyphosate + Tebuconazole | | 1 | 970 | 970.2 | 1.067 | 0.33 |
| Acetamiprid | Acetamiprid + Fluroxypyr + Azoxystrobin | | 1 | 16778 | 16778 | 39.92 | <0.0001 |
| Fluroxypyr | Fluroxypyr + Azoxystrobin | | 1 | 2355 | 2354.8 | 10.58 | 0.01 |
| Fluroxypyr | Acetamiprid + Fluroxypyr + Azoxystrobin | | 1 | 2727 | 2727.1 | 11.97 | 0.01 |
| Azoxystrobin | Fluroxypyr + Azoxystrobin | | 1 | 7520 | 7520 | 30.04 | 0.00027 |
| Azoxystrobin | Acetamiprid + Fluroxypyr + Azoxystrobin | | 1 | 8175 | 8175 | 31.99 | 0.000217 |
| Fluroxypyr + Azoxystrobin | Acetamiprid + Fluroxypyr + Azoxystrobin | | 1 | 13.7 | 13.65 | 0.255 | 0.625 |
| Acetamiprid | Acetamiprid + Tebuconazole | | 1 | 3434 | 3434 | 4.997 | 0.050 |
| Tenuconazole | Acetamiprid + Tebuconazole | | 1 | 1823 | 1822.9 | 2.038 | 0.18 |
| ***Sorghum saccharatum - Root growth inhibition*** | | | | | | | |
| Pirimicarb | Pirimicarb + Glyphosate | | 1 | 35.021 | 35.021 | 0.06569 | 0.80 |
| Pirimicarb | Pirimicarb + Glyphosate + Tebuconazole | | 1 | 1806 | 1805.7 | 6.216 | 0.032 |
| Glyphosate | Pirimicarb + Glyphosate | | 1 | 885.801 | 885.81 | 1.59885 | 0.23 |
| Glyphosate | Pirimicarb + Glyphosate + Tebuconazole | | 1 | 6111 | 6111 | 19.63 | 0.0013 |
| Pirimicarb + Glyphosate + Tebuconazole | Pirimicarb + Glyphosate | | 1 | 2344 | 2343.6 | 6.698 | 0.027 |
| Tebuconazole | Pirimicarb + Glyphosate + Tebuconazole | | 1 | 264.1 | 264.1 | 1.433 | 0.26 |
| Acetamiprid | Acetamiprid + Fluroxypyr + Azoxystrobin | | 1 | 4166 | 4166 | 18.11 | 0.0017 |
| Fluroxypyr | Fluroxypyr + Azoxystrobin | | 1 | 1 | 0.96 | 0.01 | 0.927 |
| Fluroxypyr | Acetamiprid + Fluroxypyr + Azoxystrobin | | 1 | 150.5 | 150.52 | 4.218 | 0.067 |
| Azoxystrobin | Fluroxypyr + Azoxystrobin | | 1 | 9628 | 9628 | 30.63 | 0.00025 |
| Azoxystrobin | Acetamiprid + Fluroxypyr + Azoxystrobin | | 1 | 7540 | 7540 | 29.85 | 0.00028 |
| Fluroxypyr + Azoxystrobin | Acetamiprid + Fluroxypyr + Azoxystrobin | | 1 | 127.4 | 127.4 | 1.637 | 0.23 |
| Acetamiprid | Acetamiprid + Tebuconazole | | 1 | 72 | 72 | 0.209 | 0.657 |
| Tenuconazole | Acetamiprid + Tebuconazole | | 1 | 60.3 | 60.3 | 0.239 | 0.64 |
| ***Sorghum saccharatum -- Stem growth inhibition*** | | | | | | | |
| Pirimicarb | Pirimicarb + Glyphosate | | 1 | 639.48 | 639.48 | 0.85 | 0.38 |
| Pirimicarb | Pirimicarb + Glyphosate + Tebuconazole | | 1 | 981 | 981 | 1.21 | 0.297 |
| Glyphosate | Pirimicarb + Glyphosate | | 1 | 12.20 | 12.20 | 0.021 | 0.89 |
| Glyphosate | Pirimicarb + Glyphosate + Tebuconazole | | 1 | 6 | 6.5 | 0.01 | 0.9229 |
| Pirimicarb + Glyphosate + Tebuconazole | Pirimicarb + Glyphosate | | 1 | 36.4 | 36.4 | 0.244 | 0.639 |
| Tebuconazole | Pirimicarb + Glyphosate + Tebuconazole | | 1 | 449 | 449 | 0.723 | 0.42 |
| Acetamiprid | Acetamiprid + Fluroxypyr + Azoxystrobin | | 1 | 2727 | 2727.1 | 5.299 | 0.044 |
| Fluroxypyr | Fluroxypyr + Azoxystrobin | | 1 | 4.6 | 4.56 | 0.021 | 0.89 |
| Fluroxypyr | Acetamiprid + Fluroxypyr + Azoxystrobin | | 1 | 7.8 | 7.84 | 0.043 | 0.84 |
| Azoxystrobin | Fluroxypyr + Azoxystrobin | | 1 | 4033 | 4033 | 12.78 | 0.0051 |
| Azoxystrobin | Acetamiprid + Fluroxypyr + Azoxystrobin | | 1 | 4685 | 4685 | 16.38 | 0.0023 |
| Fluroxypyr + Azoxystrobin | Acetamiprid + Fluroxypyr + Azoxystrobin | | 1 | 24.4 | 24.37 | 0.15 | 0.711 |
| Acetamiprid | Acetamiprid + Tebuconazole | | 1 | 237 | 236.7 | 0.22 | 0.65 |
| Tenuconazole | Acetamiprid + Tebuconazole | | 1 | 25 | 31.45 | 0.26 | 0.82 |
